# VectAbundance: a spatio-temporal database of *Aedes* mosquitoes observations

**DOI:** 10.1038/s41597-024-03482-y

**Published:** 2024-06-15

**Authors:** Daniele Da Re, Giovanni Marini, Carmelo Bonannella, Fabrizio Laurini, Mattia Manica, Nikoleta Anicic, Alessandro Albieri, Paola Angelini, Daniele Arnoldi, Marharyta Blaha, Federica Bertola, Beniamino Caputo, Claudio De Liberato, Alessandra della Torre, Eleonora Flacio, Alessandra Franceschini, Francesco Gradoni, Përparim Kadriaj, Valeria Lencioni, Irene Del Lesto, Francesco La Russa, Riccardo Paolo Lia, Fabrizio Montarsi, Domenico Otranto, Gregory L’Ambert, Annapaola Rizzoli, Pasquale Rombolà, Federico Romiti, Gionata Stancher, Alessandra Torina, Enkelejda Velo, Chiara Virgillito, Fabiana Zandonai, Roberto Rosà

**Affiliations:** 1 Center Agriculture Food Environment, University of Trento, San Michele all’Adige, Italy; 2 Research and Innovation Centre, Fondazione Edmund Mach, San Michele all’Adige, Italy; 3 Epilab-JRU, FEM-FBK Joint Research Unit, Trento, Italy; 4 OpenGeoHub Foundation, Doorwerth, The Netherlands; 5 Laboratory of Geo-Information Science and Remote Sensing, Wageningen University & Research, Wageningen, The Netherlands; 6 Department of Economics and Management, University of Parma, Parma, Italy; 7 Bruno Kessler Foundation, Trento, Italy; 8 Institute of Microbiology, University of Applied Sciences and Arts of Southern Switzerland (SUPSI), Mendrisio, Switzerland; 9 Centro Agricoltura Ambiente “G.Nicoli”, Crevalcore, Italy; 10 Emilia Romagna region, Bologna, Italy; 11 Fondazione Museo Civico di Rovereto, Rovereto, Italy; 12 Department of Public Health and Infectious Diseases, Sapienza University of Rome, Rome, Italy; 13 Istituto Zooprofilattico Sperimentale del Lazio e della Toscana “M. Aleandri”, Viterbo, Italy; 14 MUSE-Museo delle Scienze, Trento, Italy; 15 Istituto Zooprofilattico Sperimentale delle Venezie, Padua, Italy; 16 Institute of Public Heatlh, Tirana, Albania; 17 Istituto Zooprofilattico Sperimentale della Sicilia “A. Mirri”, Palermo, Italy; 18 Department of Veterinary Medicine, University of Bari, Bari, Italy; 19 Department of Veterinary Clinical Sciences, City University of Hong Kong, Hong Kong, People’s Republic of China; 20 EID Mediterranée, Montpellier, France

**Keywords:** Ecological epidemiology, Entomology, Ecological modelling

## Abstract

Modelling approaches play a crucial role in supporting local public health agencies by estimating and forecasting vector abundance and seasonality. However, the reliability of these models is contingent on the availability of standardized, high-quality data. Addressing this need, our study focuses on collecting and harmonizing egg count observations of the mosquito *Aedes albopictus*, obtained through ovitraps in monitoring and surveillance efforts across Albania, France, Italy, and Switzerland from 2010 to 2022. We processed the raw observations to obtain a continuous time series of ovitraps observations allowing for an extensive geographical and temporal coverage of *Ae. albopictus* population dynamics. The resulting post-processed observations are stored in the open-access database VectAbundance.This initiative addresses the critical need for accessible, high-quality data, enhancing the reliability of modelling efforts and bolstering public health preparedness.

## Background & Summary

The *Aedes* invasive mosquitoes (AIM) are a group of arthropod vectors that have attracted the interest of scientists and public health officers for the recent expansion of their geographical ranges and their capacity to transmit viruses that affect humans^1,2^. Among the different AIM, the “Asian tiger mosquito” (*Aedes* (Stegomyia) *albopictus* (Skuse, 1895)) experienced a significant range expansion in the past three decades and it is still rapidly extending northward and increasing in altitude, mediated by climate change, thermal adaptation, and the intense transportation of goods and people^2–6^. *Aedes albopictus* proved to be a competent vector of several arboviruses, including dengue, chikungunya, Zika, West Nile, eastern equine encephalitis and La Crosse viruses^7–10^, having been already responsible for several outbreaks of vector-borne diseases in Mediterranean Europe^11–15^. Besides, it also acts as a vector of filarioid nematodes, such as *Dirofilaria immitis* and *D. repens*^7^.

Given its medical importance, European, national and local guidelines exist to conduct surveys and surveillance programs to monitor *Ae. albopictus’* local abundance, population dynamics, and assess the risk of disease transmission^16,17^. These guidelines are then locally implemented according to several limiting factors, such as budgeting costs and extension of the area to monitor (e.g., Jourdain and colleagues^18^; Di Luca^19^). Among the different means for monitoring *Ae. albopictus* populations, ovitraps are cheap and efficient tools consisting of a dark container filled with water and a substrate where mosquitoes can lay eggs. Ovitraps are inspected every one to two weeks by counting the number of eggs on the substrate to retrieve information on the mosquito phenology and population dynamics. Ovitraps application for mosquito monitoring and control is widespread^20–25^ and they are used in conjunction with other mosquito control methods for comprehensive management strategies (e.g., BG-sentinels traps).

The availability of resources and funds directly affects the sampling strategies and protocols employed by the different stakeholders^26,27^: the sole Emilia-Romagna region (Northern Italy, ~4,500,000 inhabitants) refunds municipalities involved in the field monitoring activities ~70,000 euro/year (VAT included) to maintain a regional monitoring system of 755 ovitraps inspected from the end of May (week 21) to beginning of October (week 40) and pays about 20,000 euro/year to the Regional Agency for Environmental Protection for eggs counting operations^28^.

Resource limitations pose challenges to achieving comprehensive and widespread monitoring coverage^29^, restricting each monitoring effort’s temporal and spatial extent and producing gaps in data collection in certain geographical areas or during specific periods. Therefore, the collection and standardisation of these monitoring data are widely advocated^18,19,23^ and crucial to implementing passive surveillance systems^30^, i.e. modelling approaches, that can estimate and forecast the abundance and seasonality of vectors, providing undeniable support to local public health agencies. In fact, during the past two decades, statistical models have been widely used to infer the geographic distribution and phenology of *Ae. albopictus*^31^. These models have shown to be extremely useful when predicting mosquito populations/suitability estimates in areas where no observations are available due to observation paucity (gap-filling). However, the lack of standardised and accessible high-quality data can hinder the reliability of model estimates and forecasts^32^. While detailed monitoring in a specific area enhances precision in model estimates, the broader applicability of these findings may be compromised. This underscores the crucial significance of having accessible high-quality data that spans diverse spatial and temporal conditions.

In this study, we collected, processed and harmonised *Ae. albopictus* egg count observations that were sampled using ovitraps during the monitoring and surveillance activities conducted in four different countries, namely Albania, France, Italy and Switzerland from 2010 to 2022. We processed the raw observations with a temporal downscaling approach to obtain a continuous time series of ovitraps observations allowing for an extensive geographical and temporal coverage of *Ae. albopictus* population dynamics. We stored the post-processed observations in the open-access database VectAbundance. Contrary to other efforts to collect and standardize vector data (e.g. Kraemer and colleagues^33^; Braks and colleagues^34^; Uelmen and colleagues^35^) this dataset is not based on raw observations, but it proposes post-processed observations ready to be implemented for modelling applications.

## Methods

In the context of the AIM-COST COST action (https://www.aedescost.eu/), we contacted stakeholders from four European countries (Albania, France, Italy, and Switzerland) that had active monitoring and surveillance programs of *Ae. albopictus* utilising ovitraps between 2010 and 2022. We collected raw observations from 2620 ovitraps, which were already used to perform other analyses and publications (e.g. Tran and colleagues^36^; Tisseuil and colleagues^37^; Romiti and colleagues^5,38^; Da Re and colleagues^39^; Miranda and colleagues^40^; Lencioni and colleagues^25^; Ravasi and colleagues^41,42^). The collection of raw data has been extensively detailed in the aforementioned studies, thus here we provide only a brief overview of the sampling strategies and protocols utilized by various stakeholders to gather the data. Additionally, we outline the temporal downscaling methods implemented to standardize the observations.

The authors of this work signed non-disclosure agreements regarding the use and publication of the raw observations collected by various institutes. Readers needing access to the raw data should contact the original institutions through the relevant coauthors, please inspect the “contact_person” field within the VectAbundance database for relevant contact information.

### Sampling strategies

#### Albania

The Vector Control Unit, Institute of Public Health in collaboration with Local Health Care Units in Vlore and Fier, carries out the monitoring activities within the regions of Albania.

##### Ovitraps characteristics

The ovitraps involved in the mosquito monitoring program from 2014 to 2022 are made of a black plastic cylindrical vessel (9 cm height × 11 cm diameter; Ramona Ø11/H9, LuwasaⓇ Interhydro AG) with an overflow hole at 7 cm from the base. Inside the ovitraps, a deposition substrate made of germination paper (http://www.anchorpaper.com/index.php/seed-solutions) is attached using a clip. The oviposition substrate was changed from germination paper to a scratched wooden tongue depressor (1.7 × 15 cm) during the surveys of 2020–2022 in all localities except for 6 ovitraps located in the municipality of Tirana.

##### Number of ovitraps and length of the monitoring season

The municipalities of Fier and Vlore were monitored using 30 ovitraps, which were inspected weekly from late May (week 21) to late December (week 51) in 2020 and from late May (week 21) to early October (week 40) in 2021. The municipality of Lushnje was monitored biweekly using 10 ovitraps from late August (week 34) to early October (week 40) in 2020 and from late May (week 21) to late September (week 38) in 2021. The municipality of Lezhe was monitored in 2021 only, using 5 ovitraps that were inspected every two weeks from early June (week 23) to late September (week 38). The municipality of Kavaje was monitored in 2021 only, using 5 ovitraps that were inspected every two weeks from early June (week 22) to early October (week 40). The municipality of Tirana was monitored every week using 35 ovitraps from mid-May (week 20) to late December (week 51) during 2020–2022. The ovitraps inspected every two weeks were treated with VectoMax® FG based on *Bacillus thuringiensis* var. *israelensis* and *Bacillus sphaericus*. All ovitraps have been georeferenced using the WGS84 coordinate system with the EPSG code 4326.

##### Surveys and reporting

During every survey, the status of each ovitrap is assessed. If an ovitrap is found dry or overturned, the germination paper or tongue depressor is not considered, and therefore the value assigned to that ovitrap is NA. The germination paper or tongue depressor collected is delivered to the Institute of Public Health laboratories to identify and count any eggs using a stereomicroscope.

#### France

The monitoring activities within the French Riviera (Côte d’Azur) region are carried out by the Entente interdépartementale pour la démoustication du littoral méditerranéen (EIDMediterranée), a public agency for mosquitoes control in coastal wetlands.

##### Ovitraps characteristics

The ovitraps involved in the mosquito monitoring program in the municipality of Nice from 2014 to 2019 are made of a black plastic cylindrical vessel (9.5 cm height × 11 cm diameter). The deposition substrate used is a floating piece of polystyrene.

##### Number of ovitraps and length of the monitoring season

The municipality of Nice was monitored using 50 ovitraps from late May (week 21) to early October (week 40) with revisiting time every 14 days. All ovitraps have been georeferenced using the WGS84 coordinate system with the EPSG code 4326.

##### Surveys and reporting

During every survey, the status of each ovitrap is assessed. If an ovitrap is found dry or overturned, the masonite stick is not considered, and therefore the value assigned to that ovitrap is NA. The masonite sticks collected are delivered to the EIDMediterranée laboratories for egg identification and count using a stereomicroscope.

### Italy

#### Apulia

The monitoring activities within the Bari municipality, the capital city of the Apulia region, are carried out by the University of Bari.

##### Ovitraps characteristics

The ovitraps involved in the mosquito monitoring program are black plastic cups (12 cm height × 8 cm diameter) with a volume of 300 ml filled with 225 ml of tap water and equipped with a masonite stick as deposition substrate.

##### Number of ovitraps and the length of the monitoring season

The monitoring activities for *Ae. albopictus* in Bari municipality began in 2017 and were conducted sporadically, i.e. not always for more than two consecutive seasons, until 2022. Sixty-six ovitraps were placed in the municipality over 22 sites. The monitoring activities spanned from late April (week 13) to early December (week 51) with a revisiting time every 7–10 days. All the ovitraps are georeferenced in the WGS84 coordinate system EPSG:4326.

##### Surveys and reporting

During every survey, the status of each ovitrap is assessed. If an ovitrap is found dry or overturned, the masonite stick is not considered, and therefore the value assigned to that ovitrap is NA. The masonite sticks collected are delivered to the laboratory of parasitology of the University to identify and count any eggs using a stereomicroscope.

#### Autonomous Province of Trento

Within the Autonomous Province of Trento (Northeast Italy), two local stakeholders are involved in the monitoring activities of *Ae. albopictus*: the Fondazione Museo Civico di Rovereto surveys the southern part of the Province, mostly the municipalities near Lake Garda, whilst MUSE - Museo delle Scienze (MUSE) surveys the city of Trento and its surroundings.

#### Fondazione Museo Civico di Rovereto

##### Ovitraps characteristics

The ovitraps involved in the mosquito monitoring program from 2010 to 2023 are made of polypropylene (9.5 cm height × 11 cm diameter). The deposition substrate used is a masonite stick.

##### Number of ovitraps and length of the monitoring season

The number of municipalities and ovitraps employed in the Ledro e Val Lagarina areas (southern part of the Autonomous Province of Trento) was not consistent over the period 2010–2023 and varied largely every year. However, the ovitraps were always monitored from late May (week 21) to early October (week 40) with revisiting time every 14 days. All ovitraps have been georeferenced in the ETRS 1989 UTM Zone 32 N coordinate system.

##### Surveys and reporting

During every survey, the status of each ovitrap is assessed. If an ovitrap is found dry or overturned, the masonite stick is not considered, and therefore the value assigned to that ovitrap is NA. The masonite sticks collected are delivered to the MCR laboratories to identify and count any eggs using a stereomicroscope.

#### MUSE - Museo delle Scienze (MUSE)

##### Ovitraps characteristics

The ovitrap employed in the monitoring activities from 2010 to 2023 is a small black plastic container (12 cm height × 8 cm diameter, volume 400 ml) with a hole two centimetres from the edge to prevent overfilling, mimicking the preferred natural and artificial breeding sites for the species, i.e., tree-holes, rock-holes and small man-made containers. The container is filled for two-thirds with water and contains a wood or masonite rough paddle (3 cm width × 13 cm length × 0.3 cm thickness) for adult females to lay eggs on. Until 2016, diflubenzuron® 2% was added to water in each trap, but in 2017 it was replaced with the microbiological larvicide (VectoMax® FG) based on *Bacillus thuringiensis* var. *israelensis* and *Bacillus sphaericus* in granular form in dechlorinated water at a concentration of 1 ml/litre. The deposition substrate used is a masonite stick.

##### Number of ovitraps and length of the monitoring season

The municipality of Trento is monitored using 84 ovitraps from late May (week 21) to early October (week 40) with revisiting time every 14 days. All ovitraps have been georeferenced in the ETRS 1989 UTM Zone 32 N coordinate system. Data included in this work refers to 22 traps for which long-term data are available (2010–2023).

##### Surveys and reporting

During every survey, the status of each ovitrap is assessed. If an ovitrap is found dry or overturned, the masonite stick is not considered, and therefore the value assigned to that ovitrap is NA. The masonite sticks collected are delivered to the MUSE laboratories to identify and count any eggs using a stereomicroscope. The confirmation of *Ae. albopictus* identification was done by rearing eggs caught with extra masonite sticks in traps without the insecticide and with the adult collection employing BG-Sentinel traps in the same locations.

#### Emilia-Romagna

The monitoring activities within the Emilia-Romagna region are coordinated by the Local and Regional Public Health departments and are carried out operatively by the municipalities involved^21^.

##### Ovitraps characteristics

The ovitraps involved in the mosquito monitoring program from 2010 to 2022 are made of cylindrical plastic jars, black in colour, with a volume of 1.4 litres and a diameter of 11 cm (CAA14GG/CAA14G model). They are perforated at approximately 2/3 of their height to contain about 800–900 ml of solution. The used ovitraps are filled with a solution of B.t.i. (*Bacillus thuringiensis israelensis* - 1,200 UTI/mg) in dechlorinated water at a concentration of 1 ml/litre. Inside them, a deposition substrate is attached using a clip or a wooden clamp, which consists of a masonite stick (2.5 cm width × 14.5 cm length) with the rough side exposed to the water.

The ovitraps are covered by a plastic mesh with a 1 cm-sized opening, fixed along the edge to prevent contact between the solution and domestic animals, thereby reducing the risk of overturning. Additionally, the mesh prevents the accumulation of leaves or other debris inside the ovitraps, which, if allowed to ferment, could interfere with their attractiveness. As a result, the deposition substrates remain cleaner, making classification and counting easier.

##### Number of ovitraps and length of the monitoring season

Ten municipalities are monitored using 755 ovitraps from late May (week 21) to early October (week 40) with revisiting time every 14 days. All ovitraps have been georeferenced in the ETRS 1989 UTM Zone 32 N coordinate system.

##### Surveys and reporting

During every survey, the status of each ovitrap is assessed (Regional Surveillance Operative protocol^19^). If an ovitrap is found dry or overturned, the masonite stick is not considered, and therefore the value assigned to that ovitrap is NA. The masonite sticks collected are delivered to the Regional Environmental Agency (ARPAE) laboratories to identify and count any eggs using a stereomicroscope. A quality check is then performed on the egg count data following the protocol described in Carrieri and collagues^43,44^, and, if they pass the quality check, are published on the regional website (www.zanzaratigreonline.it).

#### Lazio and Tuscany

The Istituto Zooprofilattico Sperimentale del Lazio e della Toscana (IZSLT) is responsible for monitoring activities within the Lazio and Tuscany regions.

##### Ovitraps characteristics

The ovitraps involved in the mosquito monitoring program from 2017 to 2022 consisted of a 400 ml black plastic container filled with 300 ml tap water and equipped with a masonite stick (3 cm width × 15 cm length) for oviposition. The oviposition substrate was diagonally positioned with the rough side towards the centre of the container. The ovitraps were placed outdoors, at ground level, in sheltered and shaded places, and were left in the same position throughout the whole monitoring period. The ovitraps were set in urban areas, within public or house gardens, hospitals or seats of the local health service, gathering places (e.g. markets, train stations and churches), graveyards and container terminals.

##### Number of ovitraps and length of the monitoring season

The number of ovitraps employed in the Lazio and Tuscany regions varied considerably across 2017–2022. In the Lazio region, in 2017 there were 5 active ovitraps, which increased to 59 in 2018, 81 in 2019, 75 in 2020, 80 in 2021, and 85 in 2022. In the Tuscany region, the monitoring activities started in 2020 with 21 active ovitraps, which increased to 26 in 2021, and 69 in 2022. The ovitraps were monitored from late May (week 21) to early October (week 40), with revisiting time every 7 days. From early October to late May of the following year, the revising time passed to 14 days. All ovitraps have been georeferenced in the ETRS 1989 UTM Zone 32 N coordinate system.

##### Surveys and reporting

During every survey, the status of each ovitrap was assessed^38^. If an ovitrap was found dry or overturned, the masonite stick was not considered and therefore the value assigned to that ovitrap was NA. The collected masonite sticks were delivered to the IZSLT laboratory for egg identification and count. Eggs were counted under a stereomicroscope. To confirm *Ae. albopictus* identification, randomly chosen masonite sticks from each site were put in water with a source of food to allow egg hatching and larval development in adults. Adult mosquitoes were morphologically identified using the identification keys of Severini and colleagues^45^ and Ree^46^.

#### Sicily

The monitoring activities within the Sicily region are carried out by the Istituto Zooprofilattico Sperimentale della Sicilia “A. Mirri” at the laboratory of “Entomologia e controllo dei Vettori Ambientali” (EVA).

##### Ovitraps characteristics

The mosquito monitoring program spanned from January 2010 to January 2018. A second surveillance activity started in May 2021 and lasted until August 2022. The ovitraps employed consist of a black polypropylene cup with a capacity of 500 ml. An oviposition substrate consisting of a stick of masonite (2.5 cm width × 30 cm length × 0.3 cm thickness) is dipped into the water (no support is used, the stick is made of material rigid enough to self-support itself)

##### Number of ovitraps and length of the monitoring season

Five ovitraps are placed within the area of the Istituto Zooprofilattico Sperimentale della Sicilia “A. Mirri”. The ovitraps are checked with revisiting time every 7 days. All ovitraps have been georeferenced in the ETRS 1989 UTM Zone 32 N coordinate system.

##### Surveys and reporting

During every survey, the status of each ovitrap is assessed. If an ovitrap is found dry or overturned, the masonite stick is not considered, and therefore the value assigned to that ovitrap is NA. The masonite stick is delivered to the EVA laboratories to identify and count any eggs using a stereomicroscope.

#### Veneto

The monitoring activities within the Veneto region are coordinated by the Istituto Zooprofilattico Sperimentale delle Venezie (IZSVe) and operatively carried out by municipalities and Local Public Health departments.

##### Ovitraps characteristics

The ovitraps used in the mosquito monitoring programs consisted of a 400 ml black plastic container filled with 300 ml tap water and equipped with a masonite stick (3 cm width × 15 cm length) for oviposition. The ovitraps were positioned outdoors, at ground level, in sheltered and shaded areas, and they remained in the same location for the duration of the monitoring period, similar to what was previously reported for Tuscany and Lazio regions. The urban settings for the ovitraps included graveyards, container terminals, hospitals, public or residential gardens, local health service offices, and gathering places including churches, rail stations, and marketplaces.

##### Number of ovitraps and the length of the monitoring season

The number of ovitraps employed in the Veneto region varied over the years and locations according to specific surveillance plans. In the alpine area of the region, 40 ovitraps were deployed in two municipalities (Feltre and Belluno) monitored from mid-June to the end of October during 2017–2022. Other municipalities in the alpine area were monitored using three ovitraps for each municipality from mid-May to mid-October during 2017–2022. The masonite sticks were collected every two weeks. Larvicide was not applied because the larval development time in the alpine area is longer than one week due to lower temperatures.

In the continental area of the region, the Venice and Treviso airports and the commercial port of Venice were monitored from 2018 to 2022 using seven ovitraps each from mid-June to the end of October as part of the monitoring of points of entry of invasive species. The other municipalities in the continental area of the region were not monitored consistently but only for one or two years. A larvicide (*Bacillus thuringiensis israelensis* - 1,200 UTI/mg) was added to the water in ovitraps at a concentration of 1 ml/litre; the samples (masonite sticks) were collected every two weeks except in Occhiobello where no larvicide was used and sticks were collected weekly. All ovitraps have been georeferenced in the ETRS 1989 UTM Zone 32 N coordinate system.

##### Surveys and reporting

For all samplings, the status of each ovitrap was assessed. If an ovitrap is found dry or overturned or the masonite sticks are missing, NA is assigned. The collected masonite sticks were delivered to the IZSVe laboratory for egg identification and count under a stereomicroscope. In areas where the occurrence of different invasive *Aedes* species is known, randomly chosen masonite sticks from each site were put in water with a source of food to allow egg hatching and larval development. Larvae were morphologically identified using the identification keys of Severini and colleagues^45^.

#### Switzerland

The monitoring activities within the Canton of Ticino region are carried out by the Vector Ecology unit/group (SUPSI-IM-ECOVET) of the Institute of Microbiology of SUPSI. SUPSI-IM-ECOVET closely collaborates with cantonal and municipal authorities for active surveillance.

##### Ovitraps characteristics

For the mosquito monitoring program from 2020 to 2022, the ovitraps consisted of a black plastic container with a volume of 1.5 litres (Ramona Ø13/H12, LuwasaⓇ Interhydro AG) and a wooden steamed beechwood (2.5 cm width × 20 cm length × 0.5 cm thick) which function as an oviposition substrate. Containers are filled with tap water and a few grains of B.t.i. (*Bacillus thuringiensis israelensis*, Vectobac GⓇ) is added to prevent the trap from becoming a breeding site^47^.

##### Number of ovitraps and length of the monitoring season

Four municipalities are monitored using 71 ovitraps from late May (week 21) to mid-September (week 37). Samples are collected every two weeks by municipality workers. Swiss national coordinates LV95 and WGS84 coordinate systems are used as georeference for all ovitraps.

##### Surveys and reporting

For all nine samplings, the status of each ovitrap is assessed. If an ovitrap is found dry or overturned, the substrate is considered altered and eggs are counted. In case the substrate is missing, NA is assigned. The wooden slats are collected by municipality workers and are delivered to the SUPSI-IM-ECOVET laboratory to identify and count Aedine eggs using a stereomicroscope (magnification 50x).

### Data analysis

As described above, each stakeholder, i.e. public health agency or research centre, adopts different monitoring schemes (i.e., size of the ovitraps, number of ovitraps, length of the monitoring period, length of the ovitrap activation period, etc.), depending on their needs, budget, and personnel. As a result, between (but also within) European countries the monitoring schemes are highly heterogeneous^18,40^, restricting the temporal and spatial extent of each monitoring effort and producing gaps in data collection in certain geographical areas or during specific periods. Whilst this is undoubtedly problematic, some studies suggest that different type of traps can yield comparable results in terms of collected eggs (e.g., Velo and colleagues^48^).

To address this heterogeneity, we implemented a set of rules and a temporal downscaling procedure to harmonise and standardise the data. Ovitraps were generally inspected on a weekly or biweekly basis, depending on the local protocol adopted by the stakeholders. We chose the week as the fundamental temporal unit of our study, therefore, if the monitoring period was longer than one week, we performed a temporal downscaling by randomly distributing the observed egg counts throughout the trap activity period using a binomial draw with a probability equal to 1/n weeks of activation. This means that if a trap was active for 2 weeks and a total of 500 eggs were collected, the observed 500 eggs would be randomly assigned to each week with a probability p = 1/2, resulting in, e.g. 256 eggs collected during the 1st week and 244 collected during the second.

For most of the monitored ovitraps, the monitoring period spans from May (week 20) to October (week 40). Though there is some variability in the length of the monitoring period depending on the stakeholders’ resources and local protocols, the beginning and end of the monitoring year (from March to May, and from October to February in Europe) are often characterised by few or no observations. To handle missing or incomplete data and ensure consistency in analysing ovitrap egg counts throughout the years, we modified the observed data according to the following assumptions:

1. For November, December, January and February (late autumn and winter in the Northern hemisphere), if no observation data was provided, the egg count was assumed to be zero. However, if observations were available, the weekly number of eggs was calculated as the average of the observations for each month.
2. For March and April (late winter and early spring in the Northern Hemisphere), if no observation data was provided, the egg count was marked as “NA,” indicating missing or unavailable data, because under warm temperature conditions, egg hatching might already occur from March^49^.

We coded these rules into the spreader R function, which is available in the R package dynamAedes^39^ v2.2.8. To reduce variability and to standardise the sampling effort in the observed egg count over the whole area of interest, we reprojected the geographic coordinates of ovitraps with coordinate systems other than the WGS84 projection to EPSG 4326 using the st_transform function from the sf^50^ R package, and then aggregated the ovitraps within 9 × 9 km grid cells by calculating median values. Aggregating the data in this manner allowed for a more comprehensive analysis while mitigating the impact of small-scale fluctuations in the observed egg counts. The spatial resolution choice is consistent with the current resolution of the ERA5Land^51^ climatic datasets, thus allowing for a potential homogenization between the different datasets and eventually for modelling analysis.

## Data Records

**Fig. 1:**
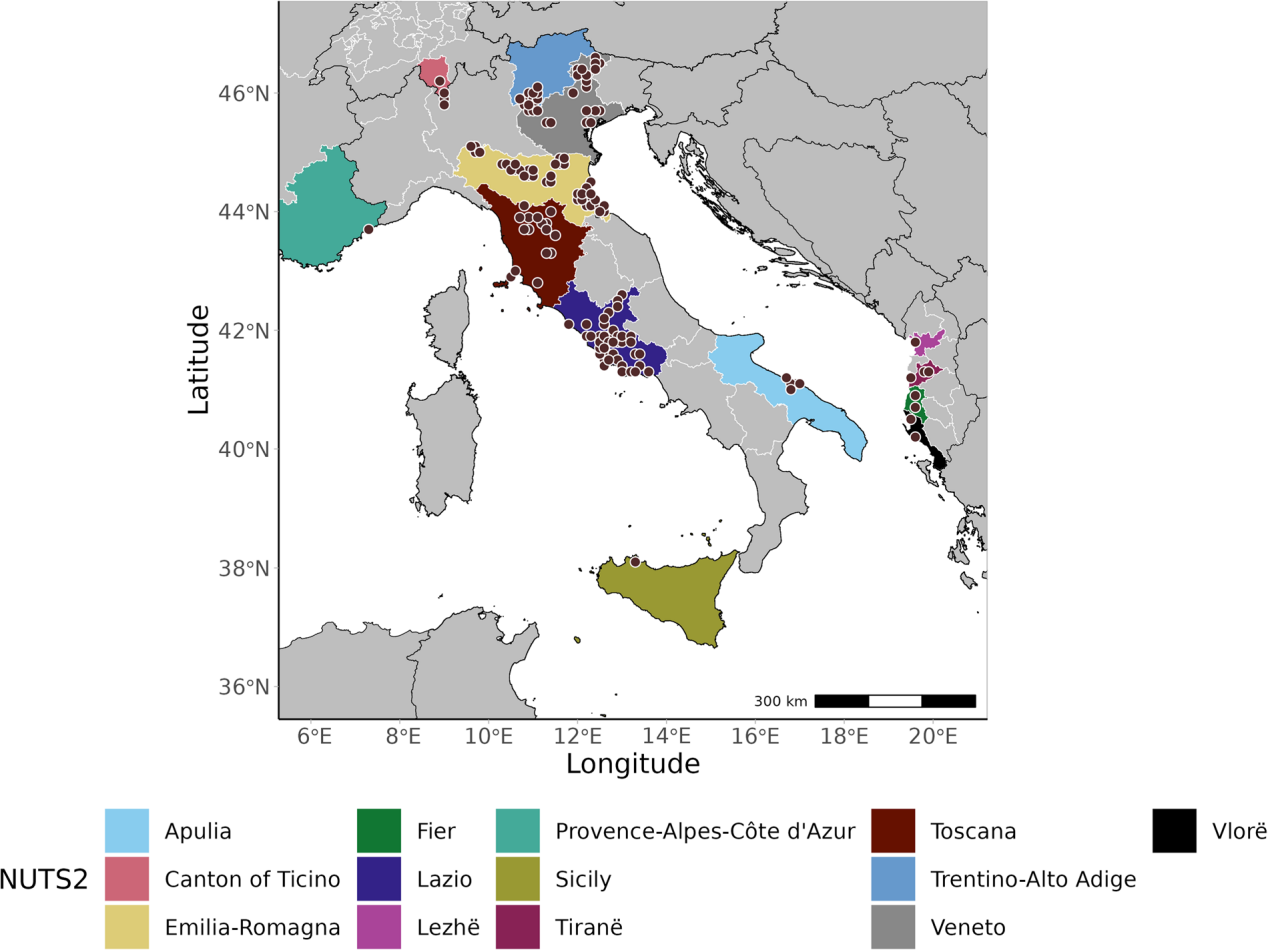
Location (brown dots) of the aggregated ovitraps at 9 × 9 km spatial resolution, with the colored polygons representing the administrative areas at the NUTS2 level where observations are available.

VectAbundance^52^ adheres to the FAIR^53^ principles and is permanently available in a Zedono repository. VectAbundance v.0.1.5 currently hosts 149 aggregated ovitrap stations, categorized by five Albanian NUTS2 administrative levels (Fier, Lezhe, Lushnje, Tirane, Vlore), seven Italian NUTS2 regions (Autonomous Province of Trento, Emilia-Romagna, Apulia, Lazio, Tuscany, Sicily, Veneto), one Swiss region (Canton of Ticino), and one French region (Côte Azur) (Table 1, Fig. 1). Most of the observations were collected during 2020–2022. Still, some NUTS2 units have a long-lasting experience of *Ae. albopictus* monitoring from 2010 (e.g. Autonomous Province of Trento and Emilia-Romagna region in Italy and Canton of Ticino in Switzerland).

**Table 1:** Aggregated ovitraps locations at the NUTS2 level and monitoring periods across various European regions.

Country	NUTS2	N. aggregated locations	Monitoring period
Albania	Fier	1 (n = 10)	2020–2021
Albania	Lezhe	1 (n = 5)	2021–2021
Albania	Lushnje	1 (n = 10)	2020–2021
Albania	Tirane	4 (n = 66)	2014–2022
Albania	Vlore	2 (n = 20)	2020–2021
France	Cote Azur	1 (n = 50)	2014–2019
Italy	Autonomous Province of Trento	15 (n = 464)	2010–2022
Italy	Emilia-Romagna	39 (n = 1451)	2010–2022
Italy	Lazio	42 (n = 162)	2017–2021
Italy	Apulia	5 (n = 81)	2012–2022
Italy	Sicily	1 (n = 29)	2021–2022
Italy	Tuscany	2 (n = 6)	2020–2022
Italy	Tuscany	14 (n = 71)	2020–2022
Italy	Veneto	11 (n = 76)	2018–2022
Italy	Veneto	8 (n = 79)	2018–2022
Switzerland	Canton of Ticino	4 (n = 71)	2010–2022

Numbers within brackets indicate the total number of deployed ovitraps.

**Fig. 2:**
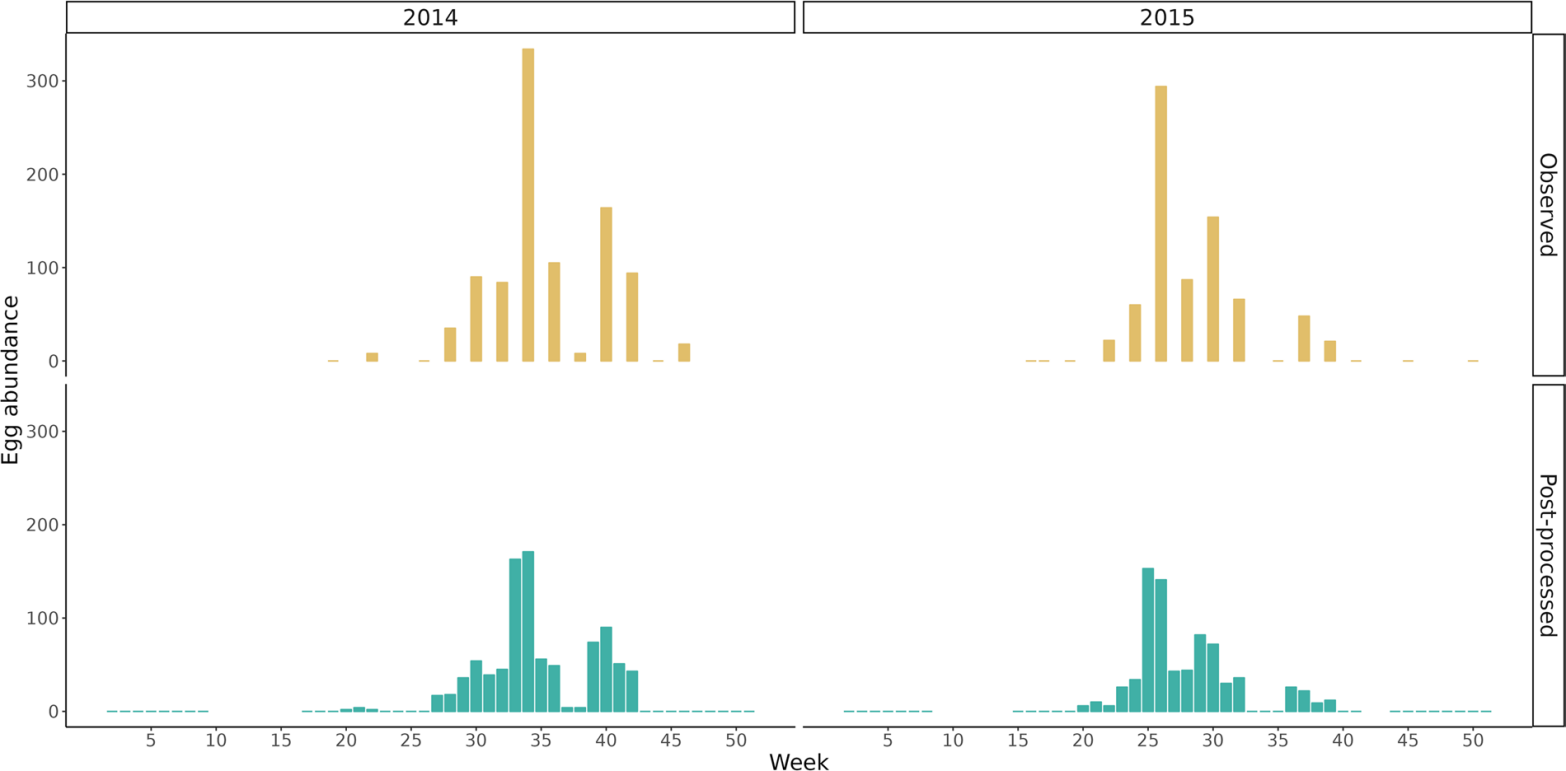
Effect of the temporal downscaling on the observed egg counts of a typical ovitrap across two sampling seasons. The general seasonal pattern remains consistent, but the observed values are now distributed throughout the ovitrap’s activity period.

The impact of post-processing, specifically the temporal downscaling, is illustrated in Fig. 2 for a typical ovitrap across two seasons. The general seasonal pattern remains consistent, but the observed values are now distributed throughout the ovitrap’s activity period, resulting in a continuous representation of vector seasonality. Additionally, the gaps at the beginning and end of the years are filled with zeros. If there were no observations available for the May-April period, which may have low *Ae. albopictus* activity^5,25,49,54,55^, those periods are marked as NA (not available).

The processed database encompasses several descriptive fields. Among them, “Canonical_name,” “kingdom,” “phylum,” “class,” “order,” “family,” “genus,” and “species” provide taxonomic information about the biological observations. The “life_stage” field specifies the life history stage of the observed species, such as eggs, larvae, pupae, or adults. “sampling_date” notes the date of trap inspection, with the count of individuals stored in the “value” field. Additional fields describe trap typology, including “trap_type,” and trap characteristics, such as “dimension”, “substrate” and “larvicide_presence”. Geographical data, including coordinates “lat” and “long” expressed in EPSG:4326, “country”, and, “region” are also included. Furthermore, there is information about the “institution” responsible for monitoring and a designated “contact_person” for relevant contact information about the accessibility to the raw data.

## Technical Validation

The ovitraps were surveyed and the eggs were analysed under the stereomicroscope by trained medical entomologists with more than a decade of experience in the field. To confirm *Ae. albopictus* identification, most laboratories randomly reared some of the eggs collected using the ovitraps and morphologically identified the larvae or the emerged adults. The procedure of temporal downscaling and spatial aggregation did not alter the observed seasonal pattern of *Ae. albopictus* egg abundance (Fig. 2). Moreover, the observed seasonal pattern well matched those reported in other studies^5,55–57^ or predicted by different modelling approaches^37,42,57^.

## Usage Notes

VectAbundance currently offers scientists, researchers, policymakers, and public health agencies access to high-quality post-processed spatio-temporal observations of *Ae. albopictus* egg abundance data. The data contained in this database are intended to be used to keep track of current, past and future records of *Ae. albopictus* presence and eggs abundance. Moreover, the database represents one of the largest openly accessible *Ae. albopictus* data sources over Europe and can be put into use for several research investigations. VectAbundance could be exploited to train and/or validate quantitative models at different geographical and temporal resolutions. Such models could be used to estimate mosquito population dynamics and abundance^39,58–60^ but also to assess the transmission risk of different *Aedes*-borne pathogens^55,61^. Despite there being few requirements for contributing data, the database could help set a reference standard for harmonising and sharing data across different countries in Europe.

## Data Availability

The code to perform the temporal downscaling is available on the CRAN at dynamAedes v2.2.8 and a tutorial illustrating how to apply the temporal dowscaling methodology is available in the article section of the package’s website (https://mattmar.github.io/dynamAedes/). The ovitraps raw observations of a specific stakeholder are available upon request to the contact person indicated in the dataset.
